# Altered Developmental and Metabolic Gene Expression in Basil Interspecific Hybrids

**DOI:** 10.3390/plants11141873

**Published:** 2022-07-18

**Authors:** Saumya Shah, Shubhra Rastogi, Divya Vashisth, Prashant Kumar Rout, Raj Kishori Lal, Umesh Chandra Lavania, Ajit Kumar Shasany

**Affiliations:** 1 Biotechnology Division, CSIR-Central Institute of Medicinal and Aromatic Plants, Lucknow 226015, India; saumyashah006@gmail.com (S.S.); shubhra.rastogi81@gmail.com (S.R.); vashisthdivya@gmail.com (D.V.); 2 Department of Phytochemistry, CSIR-Central Institute of Medicinal and Aromatic Plants, Lucknow 226015, India; pk.rout@cimap.res.in; 3 Department of Genetics and Plant Breeding, CSIR-Central Institute of Medicinal and Aromatic Plants, Lucknow 226015, India; rajkishorilal@gmail.com (R.K.L.); lavaniauc@yahoo.co.in (U.C.L.); 4 Department of Botany, University of Lucknow, Lucknow 226007, India; 5 ICAR-National Institute for Plant Biotechnology (NIPB), Pusa Campus, New Delhi 110012, India

**Keywords:** amphidiploids, flavonoid, intergenomic instability, interspecific hybrid F1, lignin, phenylpropanoid, polyploidy, RNA-seq

## Abstract

To understand the altered developmental changes and associated gene expression in inter-genomic combinations, a study was planned in two diverse yet closely related species of *Ocimum,* targeting their hybrid F1 and amphidiploids. The existing developmental variations between F1 and amphidiploids was analyzed through phenotypical and anatomical assessments. The absence of 8330 transcripts of F1 in amphidiploids and the exclusive presence of two transcripts related to WNK lysine-deficient protein kinase and geranylgeranyl transferase type-2 subunit beta 1-like proteins in amphidiploids provided a set of genes to compare the suppressed and activated functions between F1 and amphidiploids. The estimation of eugenol and methyleugenol, flavonoid, lignin and chlorophyll content was correlated with the average FPKM and differential gene expression values and further validated through qRT-PCR. Differentially expressed genes of stomatal patterning and development explained the higher density of stomata in F1 and the larger size of stomata in amphidiploids. Gene expression study of several transcription factors putatively involved in the growth and developmental processes of plants clearly amalgamates the transcriptome data linking the phenotypic differences in F1 and amphidiploids. This investigation describes the influence of interspecific hybridization on genes and transcription factors leading to developmental changes and alleviation of intergenomic instability in amphidiploids.

## 1. Introduction

All current-day flowering plants that appear to be diploid have undergone at least one round of ancient whole-genome duplication, suggesting that all angiosperms have polyploidy lineages [1,2,3]. It is estimated that up to 25–30% of angiosperms continue to remain polyploid and have not yet diploidized [4,5,6,7], and this trend is enhanced under stress [8,9]. Further, it is suggested that autopolyploidy can remodel transcriptomes and metabolomes [10,11]. As such, polyploidy is considered to be the most important event in the diversification and speciation of flowering plants [12,13]. It has been observed that polyploidy leads to increased genetic variation, offering ecological and evolutionary advantages. These advantages include robustness, increased environmental fitness and tolerance under a broad range of ecological and environmental conditions, enhanced photosynthetic efficiency, resistance to abiotic and biotic factors [7,14,15,16], enhanced productivity of secondary metabolites [17] and increased cell size, stomata and vascular cells [18,19]. However, it grants differential effect over the body size [19,20]. In addition, owing to enhanced variation and adaptation potential, polyploids could be 20% more invasive than their closely related diploids [21].

*Ocimum* is an important and the largest genus of the mint family, Lamiaceae. The genus *Ocimum,* collectively known as basil, is represented by many different varieties having incomparable curative properties and unique chemical compositions [22]. The genus shows high degrees of morphological, chemical, and genetic differences at inter- and intraspecific levels [23]. According to karyomorphological studies performed on different varieties of *Ocimum*, there exists a great variation in the chromosome number across the genus, ‘n’ number, ranging from 11–38 (http://ccdb.tau.ac.il/search/Ocimum/ (accessed on 21 June 2018)). This shows that polyploidy is a common event in *Ocimum* spp. and has played an important role in the evolution of this genus. Conventional breeding techniques and other ploidy manipulation tools have been actively used in the genetic improvement of *Ocimum* species for developing better plants, having high-yielding essential oils and other bioactive molecules [22]. The present investigation was planned on two closely related but diverse species of the model plant *Ocimum*, their interspecific hybrids and genomically doubled alloploids/amphidiploids, to elucidate developmental changes occurring during intergenomic stabilization and the associated transcriptomic insights and gene expression using high-throughput *de novo* transcriptome sequencing and digital gene expression profiling.

In this study, an interspecific hybrid F1 of *Ocimum basilicum* and *Ocimum kilimandscharicum* and their amphidiploid plants were used. Further assessment of the plants revealed that the interspecific hybrid F1 was sterile, with a smaller leaf area and taller plant height. However, the fertility was restored in genomically double amphidiploids, which had larger leaf area but shorter plant height. Moreover, amphidiploids had a thicker and stronger stem, while the stem of F1 was thinnest and weakest among all. Specifically, the trichomes and stomata in amphidiploids were bigger in size, whereas in F1 they were greater in number and smaller in size. These developmental variations in interspecific hybrids of *Ocimum* generated interest in carrying out high-throughput *de novo* transcriptome sequencing and differential gene expression profiling of parent plant (*O. basilicum* and *O. kilimandscharicum*), interspecific hybrid F1 and amphidiploid plants. This study provides a deep understanding of the parents, F1 and the genomically doubled amphidiploids and explains the underlying mechanisms responsible for the developmental changes in interspecific hybrid F1 and amphidiploids, supporting the significance of associated changes in the gene expression.

## 2. Results

### 2.1. Comparative Phenotype

**Figure 1:**
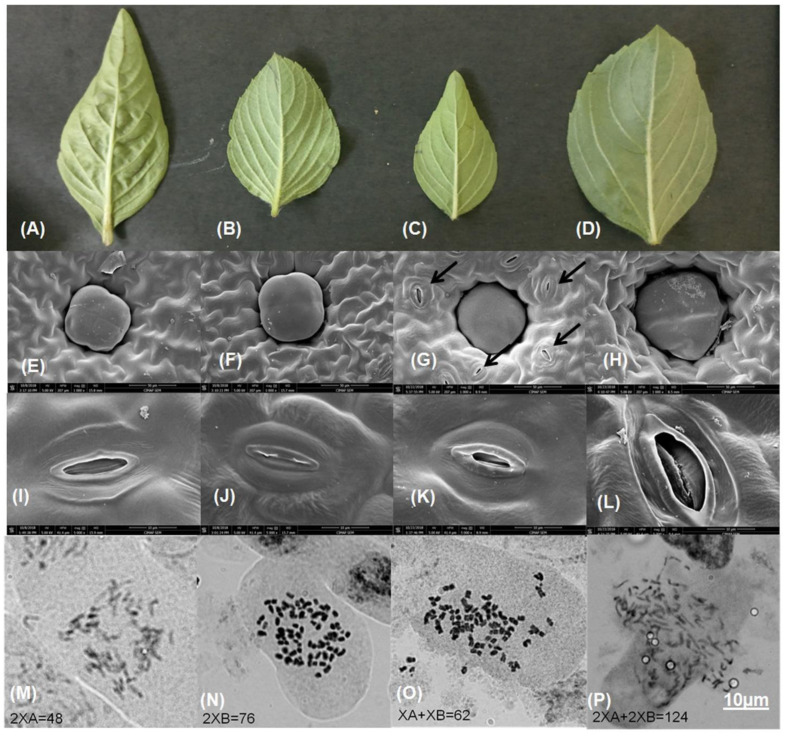
Morphological characterization of parents (OBP1 and OKP2), interspecific hybrid F1, and amphidiploids grown under same conditions. (**A**–**D**) Leaves of OBP1, OKP2, interspecific hybrid F1 and amphidiploids. (**E**–**H**) Glandular trichome of OBP1, OKP2, interspecific hybrid F1 and amphidiploids. (**I**–**L**) Stomata of OBP1, OKP2, interspecific hybrid F1 and amphidiploids. (**M**–**P**) Cytological analysis of OBP1, OKP2, interspecific hybrid F1 and amphidiploids. (**M**) Metaphase spread representing 2XA = 48 in OBP1. (**N**) Metaphase spread representing 2XB = 76 in OKP2. (**O**) Metaphase spread representing XA + XB = 62 in interspecific hybrid F1. (**P**) Metaphase spread representing 2XA + 2XB = 124 in amphiploids. Arrows in black color show density of stomata at 50 µm magnification in interspecific F1.

Morphologically interspecific hybrid F1 and its colchicine-treated amphidiploids are similar to their parent2 (OKP2). However, to identify the overall effect of polyploidization, different phenotypes, such plant height (Figure S1A–D), leaf area (Figure 1A–D), inflorescence (Figure S1E), stem diameter (Figure S1F–I), trichome density (Figure S2), trichome and stomatal length (Figure 1E–L), etc., of amphidiploids, hybrid F1 and their parents (OBP1 and OKP2) were measured (Table 1). Comparing interspecific hybrid F1 with its parent plants and amphidiploids, F1 was found to be robust, rapidly growing, vigorous and tall, while the amphidiploid was slow growing and lesser in height compared to F1. OKP2 was medium tall and slow growing, whereas OBP1 was slow growing and dwarf. Visual inspection by touching revealed that the leaf of interspecific hybrid F1 was thin, whereas the leaf of the amphidiploid was oval-shaped, broad and thick. The leaf of OBP1 was thin, while the leaf of OKP2 was thick. Similarly, the leaf area calculation revealed that the amphidiploid had~3-fold, ~1.25-fold and ~1.5-fold larger leaf area than F1, OBP1 and OKP2, respectively. In addition, the inflorescence and stem of interspecific hybrid F1 were weak, but the length of inflorescence of interspecific hybrid F1 was longer (nearly 1.5-fold) than the amphidiploid and its parents (nearly 2-fold). In addition, the stem of the amphidiploid was ~2-fold thicker than F1, OBP1 and OKP2. Besides these characteristics, the trichome density of hybrid F1 was ~2.5-fold, ~2-fold and ~1.5-fold higher than the amphidiploid, OKP2 and OBP1, respectively. Scanning electron microscopy revealed that trichomes of the amphidiploid were bigger than F1 and the parents. Similarly, the stomata of the amphidiploid were nearly 3-fold bigger than F1 and nearly 2-fold bigger than OBP1 and OKP2 (Figure 1I–L). The density of stomata was found to be greater in interspecific hybrid F1. The oil yield mg per 100 g of fresh leaf was greater in the amphidiploid (488.67 ± 22.1 mg/100 g leaves) than F1 (411.33 ± 43.57 mg/100 g leaves), OKP2 (307.33 ± 31.22 mg/100 g leaves) and OBP1 (434.67 ± 25.98 mg/100 g leaves).

### 2.2. Chromosome Number

The results obtained from root-tip mitosis of the four target plants revealed the modal somatic chromosome number to be 2n (AA) = 48 for OBP1, 2n (BB) = 76 for OKP2, n + n (A + B) = 62 for interspecific hybrid F1, and 2n + 2n (AA + BB) = 124 for amphidiploids (Figure 1M–P)**.** This suggests that the F1 hybrid and the amphidiploids constitute the genuine genomic combination of the two progenitor parents employed in the present study.

### 2.3. Transcriptome Sequencing, Assembly and Annotation

Pair-end sequencing generated nearly 14,857,263 (OBP1), 13,017,783 (OKP2), 14,266,805 (interspecific hybrid F1) and 13,955,543 (amphidiploid) reads of 101 bp average read length. After filtering and removing adapter sequences from raw reads, 14,431,491 (OBP1), 12,665,614 (OKP2), 13,866,646 (interspecific hybrid F1) and 13,479,705 (amphidiploid) high quality reads were acquired for further assembly (Table S1). The high quality reads retained after filtering raw reads contained 1,437,320,230 (OBP1), 1,261,764,356 (OKP2), 1,382,322,028 (interspecific hybrid F1) and 1,334,646,724 (amphidiploid) high quality bases. The total number of final transcripts retrieved after final assembly was 67,770 (final transcriptome length of 52 Mb with N50 value 1042) in OBP1, 73,265 (final transcriptome length of 54 Mb with N50 value 996) in OKP2, 76,917 (final transcriptome length of 58 Mb with N50 value 1008) in interspecific hybrid F1 and 76,563 (final transcriptome length of 58 Mb with N50 value in 1009) in amphidiploids. Out of 67,770 transcripts retrieved from OBP1, 52,474 were annotated and 15,296 remained un-annotated. Similarly, out of 73,265 retrieved in OKP2, 56,879 were annotated and 16,386 were un-annotated. Among the total 76,917 transcripts of interspecific hybrid F1, 60,726 were annotated and 16,191 un-annotated. On the other side, from the total 76,563 transcripts of amphidiploid, 60,954 were annotated and 15,609 remained un-annotated (Table S2).

### 2.4. Detection of Gene Expression Alterations in Interspecific Hybrid F1 and Amphidiploid

**Figure 2:**
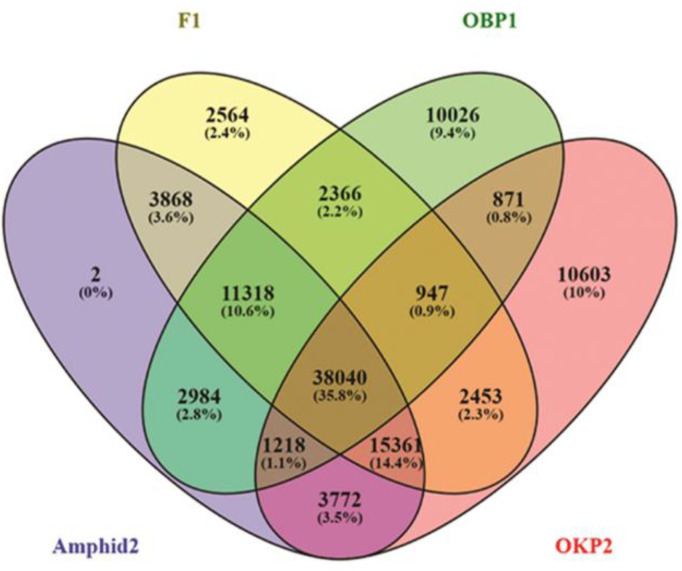
Venn diagram representing the total transcripts of parent1 (67,770), parent2 (73,265), interspecific hybrid F1 (76,917) and amphidiploids (76,563).

Gene alteration events were calculated by the occurrence of new transcripts (lacking in parents) or by the lack of some transcripts (existing in parents) in interspecific hybrid F1 and amphidiploids. For this analysis, total transcripts of parent 1 (67,770), parent 2 (73,265), interspecific hybrid F1 (76,917) and amphidiploids (76,563) were examined (Figure 2). The result of this analysis illustrated 5766 common transcripts of parents and interspecific hybrid F1 were not detected in amphidiploids. In addition to this, 6432 transcripts present in interspecific hybrid F1 and amphidiploids were missing parents. However, of these 6432 transcripts, only 3868 transcripts were common in interspecific hybrid F1 and amphidiploids. Therefore, total 8330 transcripts of interspecific hybrid F1 were absent in amphidiploids. On the other hand, only two transcripts were found to be exclusive in amphidiploids with respect to interspecific hybrid F1 and parents (OBP1 and OKP2). These alterations in gene expression may be because of gene silencing or activation or may be due to sequencing error, but here it was assumed that these transcripts were either suppressed or expressed in amphidiploids. Analysis of the annotations of these non-expressing 8330 transcripts in amphidiploids revealed that these transcripts mainly included the genes that were involved in disease resistance, primary and secondary metabolism, and cell cycle. It also included many transcription factors (“basic helix-loop-helix transcription factor”, “*MYB*/*MYB*-related”, “*MADS*-box”, “*APETALA*”, “*AP5*/*EREBP*”, “*WRKY*”, etc.), cytochrome P450s and transcripts related to methyl-CpG-binding domain-containing proteins. Moreover, there were many transcripts that were predicted as “uncharacterized or unnamed”, and many of them were also left un-annotated. In contrast, the two transcripts predicted as genes that encode WNK lysine-deficient protein kinase and geranylgeranyl transferase type-2 subunit beta 1-like proteins were exclusive to amphidiploids.

### 2.5. Comparative Gene Expression Patterns 

**Figure 3:**
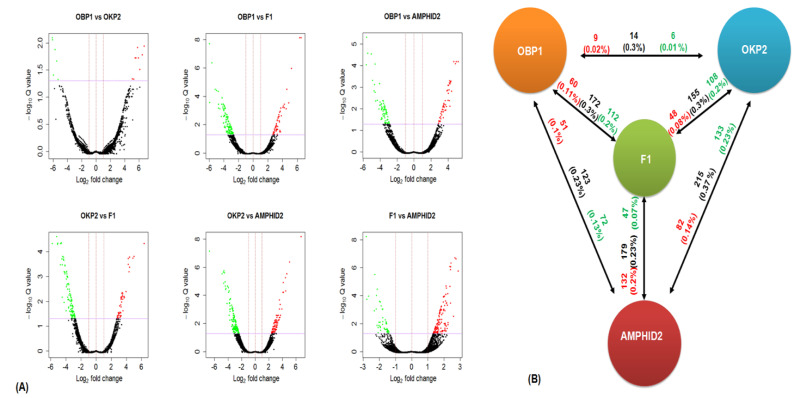
(**A**) Volcano plots showing the differentially expressed genes between the parents (OBP1 and OKP2) and their progenies (interspecific hybrid F1 and amphidiploid). Dots above the horizontal line show the differentially significant transcripts (FDR < 0.05), and the dots in red and green color above the horizontal line represent the highly significant up-regulated and down−regulated transcripts, respectively. (**B**) Diagrammatic representation of RNA−seq differential gene expression of Parent 1 (OBP1), Parent 2 (OKP2), the interspecific hybrid F1 and the colchicine−treated amphidiploid. Text written in red color indicates the total number and fraction (%) of highly significant up−regulated genes (FDR < 0.05), while text written in green color indicates the total number and fraction of (%) highly significant down−regulated genes (FDR < 0.05). The total number and fraction (%) of differentially expressed genes (FDR < 0.05) are represented by the text written in black color.

Gene expression pattern comparison between OBP1 and OKP2 resulted in 6126 up- and 5531 down-regulated transcripts. Similarly, OBP1 and F1 exhibited 4767 up- and 4187 down-regulated transcripts; OBP1 and Amphid2 reported 5007 up- and 4739 down-regulated transcripts; OKP2 and F1 showed 4204 up- and 4554 down-regulated transcripts; OKP2 and Amphid2 revealed 4110 up- and 4210 down-regulated transcripts; and 1283 up- and 1233 down-regulated transcripts were recorded from the comparison between F1 and Amphid2 (log_2_ fold change −1≥ and ≤1; Figure 3A). Among these, only nine up- and six down-regulated transcripts were found to be significant in OBP1 vs. OKP2. Similarly, 60 up- and 112 down-regulated transcripts were significant in OBP1 vs. F1. In OBP1 vs. Amphid2, 51 up- and 72 down-regulated transcripts were significant. OKP2 vs. F1 revealed 48 up- and 108 down-regulated significant transcripts, while 82 up- and 133 down-regulated transcripts were recorded as significant in OKP2 vs. Amphid2; F1 vs. Amphid2 showed 132 up- and 47 down-regulated transcripts to be significant (FDR < 0.05; BH multiple correction test; Figure 3B).

A total 179 (132 up-regulated and 47 down-regulated genes having FDR < 0.05) differentially expressed genes identified between amphidiploids and F1 hybrids (Figure S3A) were further mapped to reference canonical pathways in KEGG to determine their involvement in biological pathways, and 44 out of 179 DEGs were assigned to 46 KEGG pathways (Figure S3B). The largest cluster represented biosynthesis of secondary metabolites, with 13 members, and the second largest represented metabolic pathways, with 9 members, indicating that many genes among these DEGs were involved in the biosynthesis of secondary metabolites. Thereafter, to investigate the probable function of these DEGs, GO enrichment analysis was performed. The results of GO enrichment analysis showed that they were mainly enriched in the secondary metabolic processes, such as the sesquiterpenoid biosynthetic and metabolic process, isoprenoid biosynthetic process, and jasmonic acid metabolic process in the biological process category (Figure S4A) and were enriched in auxin:proton symporter activity, fatty-acyl-CoA reductase (alcohol-forming) activity, magnesium ion binding, lyase activity, cyclase activity, etc. (FDR < 0.05; Figure S4B).

### 2.6. DEGs Co-Regulated

After comparing all the expressed transcripts of parents and progenies, 38,040 transcripts were found commonly expressed in all four plants. In these transcripts, 71 were found to be co-regulated in OBP1 vs. Amphid2, OKP1 vs. Amphid2 and F1 vs. Amphid2 (log_2_ foldchange −1≥ and ≤1). Among these, 31 transcripts were up-regulated, and 40 transcripts down-regulated. In the 31 up-regulated transcripts, only 26 transcripts were annotated. In the 40 down-regulated transcripts, 30 transcripts were annotated (Table S3).

**Figure 4:**
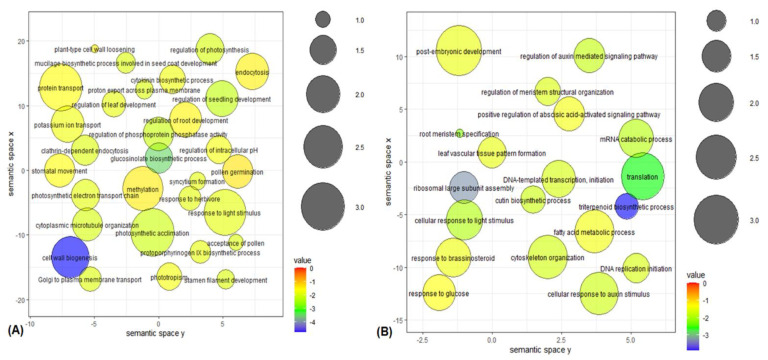
(**A**,**B**) GO enrichment analyses of DEGs co−regulated in the category of biological process (BP) terms using REVIGO. (**A**) Significantly enriched transcripts in BP GO terms related to DEGs co−upregulation (**B**) Significantly enriched transcripts in BP GO terms related to DEGs co−downregulation. Circles indicate the GO terms (plant GO slims) clustered based on semantic identities to other GO terms in ontology (larger circles depict more general terms, while adjacent circles are strongly related). Size of the circle is relative to the GO term frequency, where the colors indicate the enrichment in form of log_10_ *p* val from the AgriGO results (blue lower, red higher).

Gene ontology enrichment analysis was carried out for a set of transcripts displaying co-upregulation and co-downregulation. This analysis revealed that 75 GO terms were mainly enriched for co-upregulation and 50 GO terms for downregulation (*p* val < 0.05). In the category ‘biological processes’, cell wall biogenesis, glucosinolate biosynthesis, stomatal movement, stamen filament development, regulation of photosynthesis, intracellular pH, and seedling, leaf, and root development terms were enriched for co-upregulation. Similarly, the triterpenoid biosynthetic process, regulation of auxin-mediated signaling pathway, regulation of meristem structural organization, post-embryonic development, root meristem specification, cytoskeleton organization and cutin biosynthetic process terms were mainly enriched for co-downregulation (Figure 4A,B).

### 2.7. Verification of RNA-Seq Gene Expression Data

**Figure 5:**
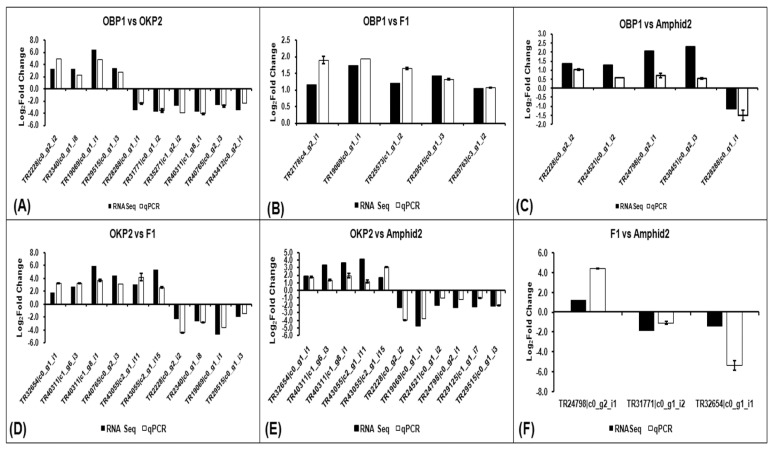
Verification of DGE results of (**A**) OBP1 vs. OKP2, (**B**) OBP1 vs. F1, (**C**) OBP1 vs. Amphid2, (**D**) OKP2 vs. F1, (**E**) OKP2 vs. Amphid2 and (**F**) F1 vs. Amphid2 with qRT−PCR. White−colored columns in the graph show the expression pattern (log_2_ fold change values) of selected transcripts of OBP1 vs. OKP2, OBP1 vs. F1, OBP1 vs. Amphid2, OKP2 vs. F1, OKP2 vs. F1, OKP2 vs. Amphid2 and F1 vs. Amphid2 using total RNA isolated from leaf tissues of OBP1, OKP2, interspecific hybrid F1 and amphidiploid through quantitative real−time PCR, while black−colored columns in the graph represent the digital gene expression values of the same transcripts.

To verify the gene expression data obtained through RNA sequencing, 23 differentially expressed transcripts common in all pairwise comparisons (OBP1 vs. OKP2, OBP1 vs. F1, OBP1 vs. amphid2, OKP2 vs. F1, OKP2 vs. Amphid2, and F1 vs. Amphid2) were randomly selected for qRT-PCR analysis (log_2_ fold change −1≥ and ≤1). Out of these 23 transcripts, the expression patterns of 11 transcripts in OKP2 vs. Amphid2, 10 transcripts in OBP1 vs. OKP2 and OKP2 vs. F1, 5 transcripts in OBP1 vs. F1 and OBP1 vs. Amphid2, and 3 transcripts in F1 vs. Amphid2 were consistent with the RNA-Seq data. Among 10 DEGs in OBP1 vs. OKP2, 4 transcripts (TR2228|c0_g2_i2, TR2340|c0_g1_i8, TR19069|c0_g1_i1, and TR29515|c0_g1_i3) were up-regulated, and 6 transcripts (TR28288|c0_g1_i1, TR31771|c0_g1_i2, TR35271|c1_g2_i2, TR40311|c1_g8_i1, TR40765|c0_g2_i3, and TR43412|c0_g2_i1) were down-regulated (Figure 5A). All 5 transcripts (TR2178|c4_g2_i1, TR19069|c0_g1_i1, TR25573|c1_g1_i2, TR29515|c0_g1_i3 and TR29763|c3_g1_i2) in OBP1 vs. F1 were found to be up-regulated (Figure 5B). However, 4 up-regulated (TR2228|c0_g2_i2, TR24521|c0_g1_i2, TR24798|c0_g2_i1, and TR30451|c0_g2_i3) and single down-regulated transcripts (TR28288|c0_g1_i1) were found in OBP1 vs. Amphid2 (Figure 5C). Of the 10 transcripts in OKP2 vs. F1, 6 were up-regulated (TR32654|c0_g1_i1, TR40311|c1_g6_i3, TR40311|c1_g8_i1,TR40765|c0_g2_i3,TR43055|c2_g1_i11, and TR43055|c2_g1_i15) and 4 (TR2228|c0_g2_i2,TR2340|c0_g1_i8,TR19069|c0_g1_i1, and TR29515|c0_g1_i3) were down-regulated (Figure 5D). Further, 5 transcripts (TR32654|c0_g1_i1, TR40311|c1_g6_i3,TR40311|c1_g8_i1, TR43055|c2_g1_i11, and TR43055|c2_g1_i15) were up-regulated, and 6 transcripts (TR2228|c0_g2_i2, TR19069|c0_g1_i1,TR24521|c0_g1_i2, TR24798|c0_g2_i1, TR29125|c1_g1_i7, and TR29515|c0_g1_i3) down-regulated in OKP2 vs. Amphid2 (Figure 5E). As shown in Figure 5F, a single transcript (TR24798|c0_g2_i1) was found to be upregulated, while two transcripts (TR31771|c0_g1_i2, and TR32654|c0_g1_i1) exhibited down-regulation in F1 vs. Amphid2. The validation of the transcriptome is shown in Figure 5.

### 2.8. General Phenylpropanoid Pathway in F1 and Amphidiploid

**Figure 6:**
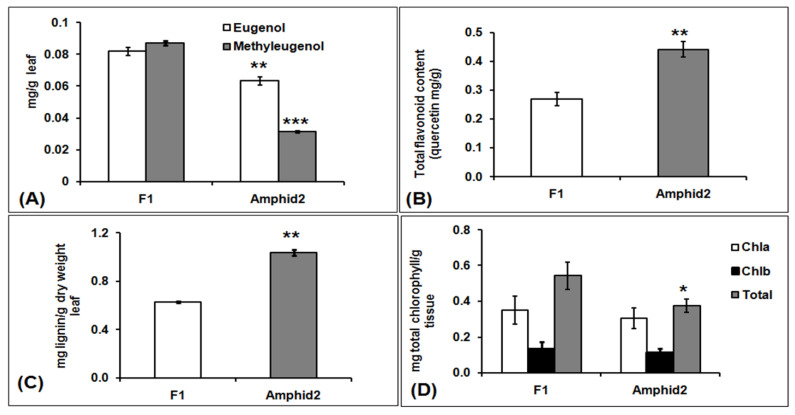
(**A**) Total amount of eugenol and methyl eugenol in the oil extracted from the 100 g of leaves of interspecific hybrid F1 and amphidiploids. (**B**) Total content change of flavonoid in interspecific hybrid F1 and amphidiploids. (**C**) Total content change of lignin in interspecific hybrid F1 and amphidiploids. (**D**) Total content change of chlorophyll metabolite in interspecific hybrid F1 and amphidiploids. The significant difference was compared between interspecific F1 and amphidiploids using the unpaired *t*-test with Welch’s correction. *** *p* < 0.0001, ** *p* < 0.001 and * *p* < 0.05.

**Figure 7:**
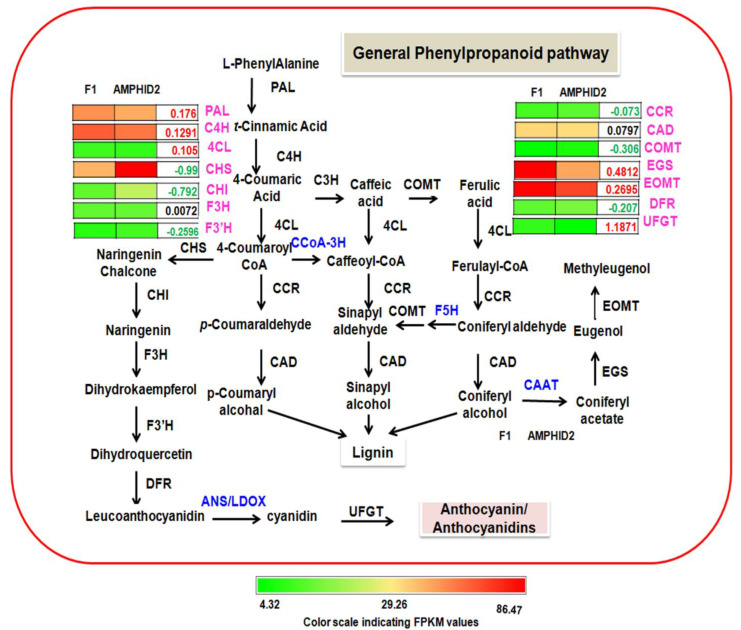
Expression analysis of general phenylpropanoid pathway genes in interspecific hybrid F1 and amphidiploids. Colored pallet represents the FPKM values, numbers in green color display down−regulated genes and numbers in red color show up−regulated genes. Genes highlighted in blue color were not annotated in the transcriptome. Abbreviations: *PAL*, phenylalanine ammonia lyase; *C4H*, cinnamate 4−hydroxylase; *4CL*, 4−coumarate: CoA, ligase; *C3H*, p−coumarate 3−hydroxylase; *COMT*, caffeoyl O−methyl transferase; *CCoAOMT*, caffeoyl−CoA O−methyl transferase; *CCR*, cinnamoyl−CoA reductase; *CAD*, cinnamyl alcohol dehydrogenase; *CAAT*, coniferyl alcohol acetyl transferase; *EGS*, eugenol synthase; *EOMT*, eugenol O−methyl transferase; *F5H*, Ferulate 5−hydroxylase; *CHS*, chalcone synthase; *CHI*, chalcone isomerase; *F3H*, flavanone 3−hydroxylase; *F3′H*, flavonoid 3′−hydroxylase; *DFR*, dihydroflavonol 4−reductase; *ANS/LDOX*, anthocyanidin synthase; *UFGT*, UDP−glucose:flavonoid 7−O−glucosyltransferase.

GC-MS profiling of essential oils revealed the prominence of eugenol and methyl eugenol in OBP1, but these phenylpropenes were not detected in OKP2 (Figure S5). However, these phenylpropenes were further identified in the oil profiles of F1 and amphidiploids. The amount of eugenol was 0.082 ± 0.003 mg/g leaf in interspecific hybrid F1 and 0.063 ± 0.002 mg/g leaf in amphidiploids, whereas the amount of methyl eugenol was 0.087 ± 0.001mg/g leaf and 0.032 ± 0.0005 mg/g leaf in interspecific hybrid F1 and amphidiploids, respectively. Further, total lignin and flavonoid content were also analyzed in interspecific hybrid F1 and amphidiploids (Figure 6A–C). The amount of flavonoid and lignin was 0.26 ± 0.023 mg/g leaf and 0.63 ± 0.005 mg/g leaf, respectively, in interspecific hybrid F1 and 0.44 ± 0.026 mg/g leaf and 1.03 ± 0.025 mg/g leaf, respectively, in amphidiploids. Thus, it was found that amount of eugenol and methyl eugenol was higher in interspecific hybrid F1, but the amount of total flavonoid and total lignin was higher in amphidiploids. In this study, the transcripts corresponding to the enzymes directly involved in the general phenylpropanoid biosynthesis pathway were identified and analyzed (Table S4). To address the question of differential biosynthesis of phenylpropanoids, lignin and flavonoids in the interspecific hybrid F1 and amphidiploids, the means of FPKM values and the values of differential gene expression (log_2_ fold change of F1 vs. Amphid2) of transcripts were used to correlate the trend of content change of phenylpropenes with its gene expression (Figure 7).

**Figure 8:**
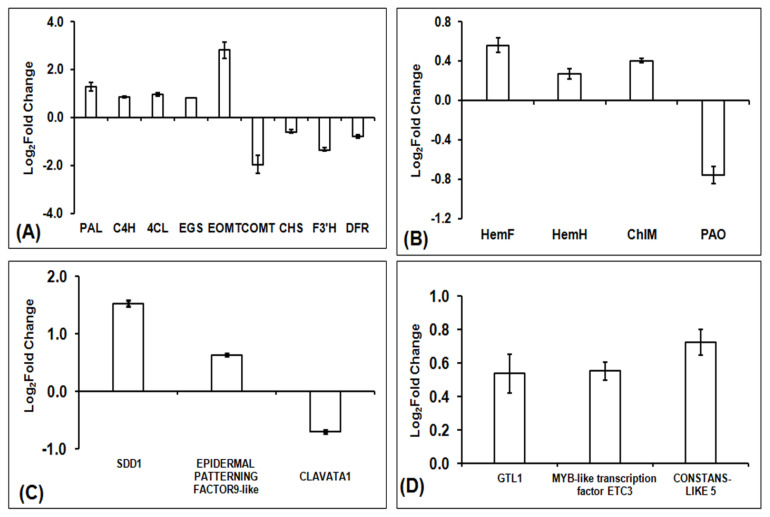
qRT−PCR analysis of selected genes of phenylpropanoid biosynthesis, chlorophyll biosynthesis, stomatal patterning and development genes and transcription factors. Columns in the graph show the expression pattern (log_2_ fold change values) of selected transcripts of (**A**) phenylpropanoid biosynthesis pathway genes, (**B**) chlorophyll biosynthesis pathway genes, (**C**) stomatal patterning and development genes and (**D**) transcription factors using total RNA isolated from leaf tissues of interspecific hybrid F1 and amphidiploids.

The average of FPKM values and differential gene expression showed that the expression of *PAL, C4H, 4CL, EGS* and *EOMT* genes were positively regulated in the interspecific hybrid F1, while the expression of *COMT* genes was negatively regulated in interspecific hybrid F1. Similarly, the expression of genes involved in flavonoid biosynthesis (*CHS, CHI, F3’H* and *DFR*) were also negatively regulated in interspecific hybrid F1. Since the values of these expressions were insignificant, to corroborate the gene expression profile of RNA-seq Data, qRT-PCR was performed for nine genes involved in general phenylpropanoid biosynthesis (Figure 8A). The results of the qRT-PCR confirm the decreased expression of *COMT CHS, F3’H* and *DFR* genes involved in the lignin and flavonoid biosynthetic pathway and the increased expression of *PAL, C4H, 4CL, EGS* and *EOMT* in interspecific hybrid F1, confirming the RNA-seq data.

### 2.9. Chlorophyll Metabolism in F1 Hybrid and Amphidiploid

**Figure 9:**
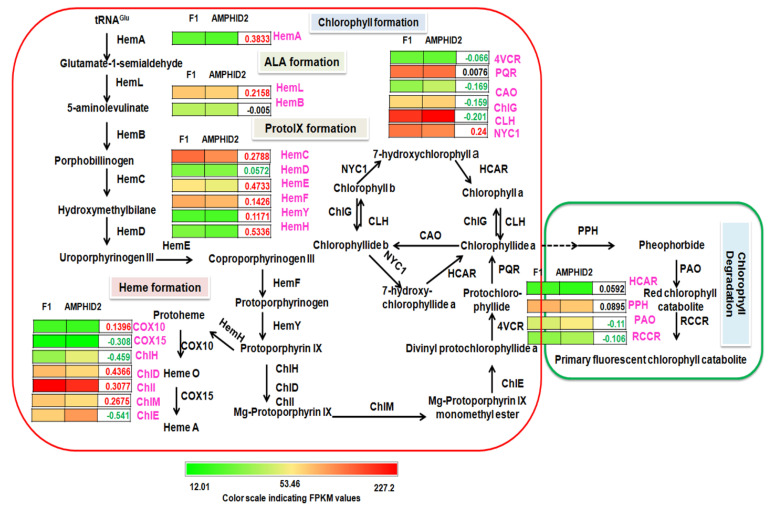
Expression analysis of chlorophyll biosynthesis pathway genes in interspecific hybrid F1 and amphidiploids. The colored pallet represents the FPKM values, numbers in green color display down−regulated genes and numbers in red color show up−regulated genes. Abbreviations: *HemA*, Glutamyl−tRNA reductase; *HemL*, Glutamate−1−semialdehyde 2, 1−aminomutase; *HemB*, Porphobilinogen synthase; *HemC*, Hydroxymethylbilane synthase; *HemD*, Uroporphyrinogen−III synthase; *HemE*, Uroporphyrinogen decarboxylase; *HemF*, Coproporphyrinogen−III oxidase; *HemN*, Oxygen-independent coproporphyrinogen−III oxidase; *HemY*, Oxygen−dependent protoporphyrinogen oxidase; *HemH*, Ferrochelatase; *COX10*, Protoheme IX farnesyltransferase; *COX15*, Cytochrome c oxidase assembly protein subunit 15; *ChlH*, Magnesium chelatase subunit H; *ChlD*, Magnesium chelatase subunit D; *ChlI*, Magnesium chelatase subunit I; *ChlM*, Magnesium protoporphyrin IX methyltransferase; *ChlE*, Magnesium−protoporphyrin IX monomethyl ester (oxidative) cyclase; *4VCR*, Divinyl chlorophyllide a 8−vinyl−reductase; *POR*, Protochlorophyllide reductase; *CAO*, Chlorophyllide a oxygenase; *ChlG*, Chlorophyll synthase; *CLH*, Chlorophyllase; *NYC1*, Chlorophyll (ide) b reductase; *HCAR*, 7−Hydroxymethyl chlorophyll a reductase; *PPH*, Pheophytinase; *PAO*, Pheophorbide a oxygenase; *RCCR* Red chlorophyll catabolite reductase.

The results of chlorophyll estimation showed that the amounts of Chla and Chlb and the total chlorophyll were 0.35 ± 0.045 mg/g, 0.13 ± 0.040 mg/g and 0.54 ± 0.054 mg/g, respectively, in interspecific hybrid F1 and 0.30 ± 0.033 mg/g, 0.11 ± 0.021 mg/g and 0.37 ± 0.026 mg/g in amphidiploids. Here, it was observed that the amounts of Chla and Chlb and the total chlorophyll contents were higher in the interspecific hybrid F1 than its amphidiploid (Figure 6D). To find the probable reason for this content change, transcripts related to 27 classic enzymes involved in the chlorophyll metabolic pathway were analyzed (Table S5). FPKM values and log_2_ fold change values for transcripts in interspecific hybrid F1 and amphidiploids were averaged for further analysis. Based on these identified transcripts, a proposed chlorophyll metabolic pathway in interspecific hybrid F1 and amphidiploids was constructed (Figure 9). The difference in the content change of Chla, Chlb and total chlorophyll was correlated with the average FPKM and the log_2_ fold change values of the transcripts related to 27 classic enzymes involved in the chlorophyll metabolism. The trend of change in expression based on the average FPKM and fold change values of transcripts related to of the main enzymes (HemA, HemL, HemC, HemE, HemF, HemY, HemH and COX10) involved in the Ala, Proto IX and Heme and the key enzymes (Chl, ChlI, ChlM and ChlE) of chlorophyll formation showed increased expression in interspecific F1 compared to amphidiploids. On the other hand, transcripts related to several enzymes (PAO and RCCR) involved in the chlorophyll degradation showed increased expression in amphidiploids compared to interspecific hybrid F1. These results clearly demonstrate that the enzymes involved in chlorophyll biosynthesis (Ala, Proto IX, Heme and Chlorophyll formation) were positively regulated in interspecific hybrid F1. In contrast, the transcripts related to the enzymes involved in chlorophyll degradation (PAO and RCCR) were negatively regulated in the interspecific hybrid F1. However, the differential gene expressions of these genes were not significant. Therefore, to validate the gene expression profile of RNA-seq data, qRT-PCR was performed. The results of qRT-PCR revealed that expression of *HemF*, *HemH* and *ChlM* transcripts were up-regulated, whereas the expression of *PAO* was down-regulated, in interspecific hybrid F1 (Figure 8B). Hence, these results correlate with the results obtained from the chlorophyll estimation.

### 2.10. Differentially Expressed Stomatal Patterning and Development Genes

Higher stomatal density in interspecific hybrid F1 and larger stomatal size in amphidiploids were two peculiar characteristics observed through anatomical analysis. On investigating the transcriptome data, several transcripts putatively associated in the stomatal patterning and development were identified in OBP1, OKP2, F1 and amphid2 (Table S6). The identified transcripts included *TOO MANY MOUTHS (TMM)*, *EPIDERMAL PATTERNING FACTOR 4 (EPF 4)*, *CLAVATA1/CLAVATA1-like, CLAVATA3/CLAVATA3*-like, *ERECTA/ERECTA*-like, various types of mitogen-activated protein kinase (MAPK), such as *MAPK9/MAPK9-like, MAPK15/MAPK15-like, MAPK19/MAPK19-like, MAPK20/MAPK20-like*, mitogen-activated protein kinase homolog *MMK1-like*, mitogen-activated protein kinase homolog *NTF3,* mitogen-activated protein kinase kinase kinase *MAPKK2/MAPKK2-like, MAPKK5/MAPKK5-like, MAPKK6/MAPKK6*-like, MAP Kinase Kinase Kinase *YODA (YDA)*, *EPIDERMAL PATTERNING FACTOR*-*like 9* (*EPF9*)/*SDD1*, *CLAVATA2*, mitogen-activated protein kinase *MAPK4/MAPK4*-*like*, *MAPK7/MAPK7*-*like*, *MAPK10/MAPK10*-like, *MAPK16/MAPK16*-like, mitogen-activated protein kinase homolog *NTF4*-*like*, mitogen-activated protein kinase homolog *MMK2*-like, mitogen-activated protein kinase kinase kinase *MAPKKK1/MAPKKK1*-like, *MAPKKK3/MAPKKK3*-like, etc. Among these, transcripts, related to mitogen-activated protein kinase 3 (TR39326|c0_g2_i11), *SDD1* encoding subtilisin-like protease (TR43953|c5_g9_i3, TR43953|c5_g9_i2, TR21221|c0_g1_i2, TR43953|c5_g10_i5 and TR43953|c5_g1_i2) and *EPIDERMAL PATTERNING FACTOR*-*like 9* (TR29381|c1_g1_i1) were found to be up-regulated in F1 vs. Amphid2. In contrast, the transcripts related to mitogen-activated protein kinase kinase kinase 2-like (TR28534|c1_g3_i1), mitogen-activated protein kinase 16 (TR23548|c0_g2_i1), *CLAVATA1* (TR39567|c0_g1_i3, and TR39567|c0_g1_i1) and *CLAVATA1*-like (TR36841|c0_g2_i1, and TR39567|c0_g3_i1) were found to be down-regulated in F1 vs. Amphid2 (log_2_ fold change −1≥ and ≤1). These results were further validated through qRT-PCR, in which *SDD1* (TR43953|c5_g1_i2) and *EPIDERMAL PATTERNING FACTOR-like 9* (TR29381|c1_g1_i1) were up-regulated and *CLAVATA1* (TR39567|c0_g1_i3) was down-regulated in interspecific hybrid F1 (Figure 8C). These results suggest that *SDD1*, *EPIDERMAL PATTERNING FACTOR-like 9* and *CLAVATA1* could be critical genes, possibly responsible for the higher stomatal density and larger stomatal size in interspecific hybrid F1 and amphidiploids, respectively.

### 2.11. Transcription Factors

TFs play an important role in the growth and development of all organisms. Based on transcription factor database PlantTFDB 4.0 (http://planttfdb.cbi.pku.edu.cn/ (accessed on 11 October 2019)) [24] and a literature search, different TFs were identified in OBP1, OKP2, F1 and Amphid2 (Table S7). Further, to investigate the role of TFs in in the altered phenotype of interspecific F1 and its improvement in amphidiploids, differentially expressed TFs between interspecific F1 vs. amphidiploids were analyzed. Of these, *MYC2* (TR33191|c0_g7_i1 and TR4095|c0_g2_i1), *MYB*-*like* transcription factor *ETC3* (TR7473|c0_g1_i3 and TR7473|c0_g1_i6), *bHLH35* (TR25010|c0_g1_i1 and TR4050|c0_g1_i1), *bHLH13*-like (TR43807|c0_g5_i3) and *bHLH60*-*like* (TR36728|c0_g6_i3), ethylene-responsive transcription factor *ERF118*-like (TR43164|c6_g1_i1) and ethylene-responsive transcription factor-like protein *At4g13040* isoform X3 (TR38204|c1_g5_i4), *WRKY* 1-like isoform X2 and *WRKY40* (TR42259|c2_g4_i1 and TR37841|c0_g6_i6), zinc finger *CCCH* 18 isoform X1 (TR41635|c0_g6_i4) and *CONSTANS-LIKE 9*-like (TR32691|c1_g3_i6), trihelix transcription factor *GTL1* isoform X2 (TR39746|c0_g1_i4), *TCP10*-*like* (TR39039|c0_g2_i3), *MYB*-related transcription factor (TR30416|c0_g1_i2 andTR30416|c0_g1_i1), transcription factor *BEE 2* (TR36728|c0_g3_i3) and *GATA* transcription factor 1-like isoform X1 (TR34578|c2_g8_i1) were up-regulated (log_2_ fold change −1≥ and ≤1). On other hand, *zinc finger CCCH 20-like* and *23* (TR40708|c1_g2_i1, TR40708|c1_g2_i10, TR40708|c1_g2_i13, TR40708|c1_g2_i7, TR35004|c0_g2_i2, TR40708|c1_g1_i1), *BEL1*-*like* 1 (TR42221|c2_g1_i1) and 4 (TR42290|c0_g2_i3), *GATA* transcription factor *11*-like isoform X2 (TR35761|c0_g4_i1) and 28 (TR40706|c0_g3_i2), *myb* family transcription factor *APL* isoform X4 (TR38890|c0_g6_i1), *306-like* (TR35145|c0_g2_i1), *R2R3-MYB* (TR34760|c2_g2_i1) and *MYB*-related transcription factor (TR2394|c0_g2_i2), *BES1*/*BZR1* homolog protein 4-like (TR38770|c0_g1_i7 and TR38770|c0_g1_i9), *CONSTANS*-*LIKE 5-like* (TR35873|c0_g2_i2),*CONSTANS*-*LIKE 9* (TR43266|c0_g12_i1), *CONSTANS*-LIKE 16/16-like (TR32758|c2_g2_i8 and TR34433|c1_g5_i2), transcription factor *LHW*-*like* (TR43885|c1_g11_i1 and TR25379|c0_g2_i1), *NAC* 2 (TR29000|c0_g2_i5), *auxin response factor 17* isoform X1 (TR44558|c1_g6_i1), *FAR1-RELATED SEQUENCE 7*-like (TR22981|c0_g1_i1), *WRKY20* (TR8059|c0_g2_i1), *MADS*-box protein *SOC1*-like isoform X2 (TR38712|c2_g1_i3), *bHLH143*-like (TR40826|c1_g4_i4), *PHD* finger *ALFIN*-*LIKE* 3-like isoform X2 (TR36879|c0_g7_i4) and *GTE10*-*like* (TR43443|c1_g10_i1) were found to be down-regulated (log_2_ fold change −1≥ and ≤1). Among these differentially expressed TFs, trihelix transcription factor *GTL1* isoform X2 (TR39746|c0_g1_i4), *MYB*-like transcription factor *ETC3* (TR7473|c0_g1_i3 and TR7473|c0_g1_i6) and zinc finger *CONSTANS*-*LIKE 5-like* (TR35873|c0_g2_i2) were found to be up-regulated in qRT-PCR (Figure 8D), which further corroborates the transcriptome data.

## 3. Discussion

### 3.1. Gene Silencing and Activation in Interspecific Hybrid F1 and Amphidiploids

Chromosome doubling after interspecific or intergeneric hybridization leads to the development of new allopolyploid species [25]. Newly formed allopolyploids must overcome the reduced fertility (occurring due to improper chromosome pairing and segregation) to prove themselves as a successful species and the associated alteration of gene expressions [26,27]. These alterations in gene expression may occur because of gene silencing and activation. However, the type of gene affected and the probable mechanism involved in the ploidy regulation of gene expression are still challenging [28]. Very few works have addressed such responses in gene alterations in the eukaryotic system. In yeast, Galitski et al., 1999 [29], showed that ploidy-regulated activation and silencing of genes were mainly related to cell growth and development. In a newly synthesized wheat allotetraploid, the silenced/lost genes included rRNA genes and genes involved in metabolism, disease resistance, and cell cycle regulation; the activated genes were of known function, and all were retroelements [25]. In this study, it was found that disappearance/suppression of genes in amphidiploids from interspecific hybrid F1 was mainly associated with the disease resistance, primary and secondary metabolism, and cell cycle. Suppression of many transcription factors like basic helix-loop-helix transcription factor, “*MYB*/*MYB*-related”, “*MADS*-box”, “*APETALA*”, “*AP5/EREBP*”, “*WRKY*” and cytochrome P450 might have helped interspecific hybrids to overcome the reduced fertility in amphidiploids. The silencing of genes encoding methyl-CpG-binding domain-containing proteins in amphidiploid plants indicated that the formation of amphidiploids is associated with epigenetic changes. This suggests that the hybridization and allopolyploidy causes rapid changes in gene structure and expression, which contributes to the novel type of expression profiles. Further investigation of unknown/uncharacterized gene suppression/lost expression in amphidiploids could provide a better understanding of the genes affected and the mechanism involved in the ploidy regulation of gene expression upon chromosome doubling.

### 3.2. Higher Amount of Eugenol and Methyl Eugenol in Interspecific Hybrid F1 Is Possibly Associated with Reduced Lignin and Flavonoid Biosynthesis

Contribution of a robust lignin biosynthesis mechanism in the processes of heterosis and enhanced adaptability in amphidiploids cannot be ignored. Lignin biosynthesis is shared by the general phenylpropanoid pathway [30], which requires deamination of phenylalanine, successive hydroxylation, and *O*-methylation of the aromatic ring, followed by the conversion of the side-chain carboxyl to an alcohol group [31]. In this study, the higher amount of phenylpropenes (eugenol and methyl eugenol) in interspecific hybrid F1 (and not in amphidiploid) is possibly due to the down-regulation of the genes involved in the production of lignin and flavonoids, diverting some flux towards the production of a higher amount of phenylpropenes in interspecific hybrid F1. Earlier studies on the modification of lignin plants suggest that the down-regulation of the lignin pathway alters the carbon flux within the phenylpropanoid pathway and indirectly influences the production of other secondary metabolites [32]. For example, down-regulation of *COMT* to low activity levels reduces lignin content by 30% in alfalfa and maize and 17% in poplar [33]. Similarly, in *Petunia hybrida*, up-regulation of the expression of cinnamate-4-hydroxylase (*C4H*) increased the flux through the phenylpropanoid pathway [34]. Rastogi et al., 2013 [35], also reported that transient down-regulation using RNA interference (RNAi) suppression of *OS4CL* in *O. sanctum* leaves caused a reduction in leaf eugenol content. Down-regulation of the chalcone synthase (*CHS*) gene in flax also showed decreased lignin synthesis and significant plant morphology, modulating the flux towards tannins [36] The suppressed differential gene expression of *COMT*, *CHS* and *DFR* genes was positively correlated with a reduced content of lignin and flavonoids in interspecific hybrid F1.

### 3.3. Enhanced Chlorophyll Biosynthesis in Interspecific Hybrid F1

The increase in chlorophyll content serves as an indicator of hybrid vigor, as it is believed that an increase in chromosome number tends to increase the number of chloroplasts in cells and hence increases the chlorophyll content [37,38,39]. However, this tendency is not always anticipated, such as in *Atriplex confertifolia*, which remains constant in plants of different ploidy levels [40]. In the present investigation, the increased chlorophyll content (chla, chlb and total chlorophyll) of hybrid F1 compared to amphidiploids correlates with respective FPKM, differential gene expression values and qRT-PCR. The increased level of chlorophyll in F1 also correlates with the increased expression of chlorophyll biosynthetic genes and decreased expression of the degradation genes in contrast to amphidiploids. Relative chlorophyll content was found to be similar in haploid, diploid and tetraploid plants of *Ricinus communis* [41]. However, while the literature does not provide any concrete foundation of this suggestion, the result of qRT-PCR of *HemF*, *HemH*, *ChlM* and *PAO* genes involved in chlorophyll biosynthesis justifies the enhanced chlorophyll content in interspecific hybrid F1.

### 3.4. Effect of Hybridization on Stomatal Patterning and Development Associated Genes

Stomatal density, guard cell length and stomatal plastid number have frequently been used as morphological markers to test ploidy levels in plants [42]. In *Coffea canephora*, significant differences in stomatal frequency and guard cell length were noticed between diploids and tetraploids [18]. In this work, it was found that stomatal density in interspecific hybrid F1 (2n = 62) was nearly two-fold higher than its amphidiploids (2n = 124). In contrast to this, the length of stomata of amphidiploids was found to be nearly three-fold greater than its interspecific hybrid F1. Therefore, to understand the genetic basis underlying such variation in stomatal frequency and stomatal length upon change in ploidy levels, several genes putatively involved in stomatal patterning and development were analyzed. In *Arabidopsis*, several components in the series of stomatal patterning and development have been identified, which include putative receptor *TOO MANY MOUTHS (TMM)* gene, Erecta-gene family, *CLAVATA*, stomatal density and distribution 1 (*SDD1*), and several *EPIDERMAL PATTERNING FACTORs* (*EPFs*) [43]. In addition, the *SDD1*-like protease gene (which shares high level of identity with *SDD1*) is concerned with epidermal development. In addition, the downstream MAP kinase signaling cascade negatively regulates stomatal development. Similarly, a defect in the *YODA* gene (*YDA*), a putative *MAP kinase kinase kinase (MAPKKK*), results in an excessive number of clustered stomata [44,45,46].

In this study, the transcripts associated with stomatal patterning and development, such as *EPIDERMAL PATTERNING FACTOR-like 9* (*EPF9*), and *SDD1* gene encoding subtilisin-like protease, *CLAVATA2,* were upregulated, indicating the involvement of these genes in the higher number of stomata in interspecific hybrid F1. In contrast, down-regulation of *CLAVATA2* in interspecific hybrid F1 implied the involvement of these transcripts in determining the higher number but smaller size of stomata in interspecific hybrid F1 and vise-versa in amphidiploids.

### 3.5. Differentially Expressed Transcription Factors

Earlier studies on model plants such as *Arabidopsis* suggest the involvement of *MYB*, *MAD*, *NAC*, *WRKY*, *bHLH* and *bZIP* transcription factors in plant growth and development processes [47]. In *Arabidopsis*, the majority of TFs belonging to the MADS box family were specifically involved in floral developmental processes [48]. In this study, the DGE of *SOC1*-like MADS-box was down-regulated in interspecific hybrid F1. In strawberry, *SOC1*- represses the flowering and promotes vegetative growth [49]. Similarly, *CONSTANS* genes also promote flowering in *Arabidopsis* [50]. Here, the DGE of *CONSTANS 5*-like transcript was found to be up-regulated, the over-expression of which promotes flowering in short day grown *Arabidopsis* [51]. *MYB* transcription factors are regarded as a master regulator of the phenylpropanoid pathway, which is also implicated in the formation of trichome in *Arabidopsis* [52,53]. Up-regulation of MYB-related transcription factor and *MYB-like* transcription factor *ETC3* could be indicative of the higher amount of phenylpropenes (methyl eugenol and eugenol) and smaller size of trichomes in interspecific hybrid F1. In *Arabidopsis*, *MYB-like* transcription factor *ETC* acts as a negative regulator of trichome development [54]. In addition, previous studies have revealed the involvement of *GATA* transcription factors in chloroplast development, seed germination, flower development, response to light and lateral root initiation identity [55]. Thus, differential expression of several TFs related to *GATA* proves its potential role in the regulation of chlorophyll biosynthesis and weaker phenotype in interspecific hybrid F1. In this study, transcripts related to trihelix transcription factor *GTL1* isoform X2 were also found to be up-regulated in interspecific hybrid F1. Earlier studies in *Arabidopsis* showed the involvement of trihelix transcription factor *GTL1* in the regulation of water use efficiency and drought tolerance by modulating stomatal density via trans-repression of *SDD1* [56]. It also regulates the ploidy-dependent cell growth in the *Arabidopsis* trichome [57]. Although the above discussion suggests the involvement of TFs in the phenotypic variability and metabolic variation in these two hybrids, in-depth functional characterization could bring out a clear understanding of regulation of these TFs in developmental alteration and metabolic variation in interspecific hybrid F1 and amphidiploids.

## 4. Materials and Methods

### 4.1. Plant Materials

The two well-identified clones of the *Ocimum* species, namely (i) *Ocimum basilicum* L. OBP1 i.e., parent 1, and (ii) *Ocimum kilimandscharicum* Gürke–OKP2 i.e., parent 2, available at the CSIR-Central Institute of Medicinal and Aromatic Plants, Lucknow 226015, India, were used as the starting material. An interspecific hybrid was produced between the two target species by hand pollination, taking OBP1 as the female parent. Interspecific hybrid F1 was raised from the seeds obtained from the fertilized ovules borne on the female parent. This hybrid thus obtained was seed sterile. Therefore, it was multiplied vegetatively to raise its clonal progenies. Shoot tips of the fast-growing hybrid at 10–12 leaf stage was administered with 0.2% aqueous solution of Colchicine (Sigma Aldrich, St. Louis, MO, USA) following cotton swab and the intermittent colchicine dropping method for 24 h. Further, colchicine treatment was terminated by eliminating the cotton swab, and the colchicine treated shoots were washed carefully with the help of a water sprayer; shoots were permitted to grow naturally. The colchicine-affected shoots were excised after growth of 10–12 leaf whorls and multiplied vegetatively to raise the amphidiploids (Amphid 2). The amphidiploids were seed fertile.

Parents (OBP1 and OKP2), interspecific hybrid F1 and Amphid 2 were cloned from cutting and planted in the BT-2 field of CSIR-CIMAP, Lucknow. Mature plants of a six-month-old age grown under the same environmental conditions were used for all analysis provided in this report. Apical leaves of ten individual plants were taken for the trait measurement and phenotype assessments. The leaves of these ten plants were pooled together to make three biological replicates for biochemical analyses.

### 4.2. Phenotypic Changes and Their Statistical Analysis

Plant heights of all four plants were measured, taking inflorescence as a starting point, until the bottom of the plant was above the soil. The main stem of all four plants was used to measure the stem diameter. Similarly, the inflorescences from each plant were taken above from the leaf part to calculate its length. The leaf area, trichome density, length of trichome and length of stomata were calculated from the apical leaves of the mature plants (six months old). The digital pictures of each part of all four plants, leaves and inflorescence (OBP1, OKP2, F1 and Amphid2) depicting the phenotypic changes were taken. Glandular trichome density was observed under 40× magnification using a compound microscope (Leica DM750) and scanning electron microscope. Leaf area was calculated using ImageJ software.

### 4.3. Scanning Electron Microscopy

Leaves of all four plants (OBP1, OKP2, F1 and Amphid2) were cut into small pieces and fixed in 100% methanol for 10 min. Fixed leaves were dehydrated through acetone series (30, 50, 70, 80, 90 and 100%), each for 10 min and then dried using a critical point dryer (K850 from Quorum Technology, Lewes, UK). After this, the dried leaves were mounted on the scanning electron microscopy stubs for sputter-coating with gold using Q150TES from Quorum Technology, UK, and observed under a scanning electron microscope (Quanta 250, FEI, Thermo Fisher Scientific, Waltham, MA, USA).

### 4.4. Chromosome Count

To ascertain the chromosome status of progenitor parents, their F1 hybrid and the amphidiploids, the somatic chromosome analysis procedure was performed on the four sets of plants. For this, the shoot cuttings were planted in sand, and the fast-growing roots emerging from the nodes were excised and then pretreated in saturated aqueous solution of *para*-dichlorobenzene for three hours at 12–14 °C, followed by thorough washing in water and fixation overnight in Carnoy’s solution (6:3:1, Absolute alcohol: Chloroform: Acetic Acid). Root tips were stained overnight at 37 °C in 2% Aceto-Orcein: 1N HCl. Fixed root tips were squashed in 45% acetic acid to observe chromosome count under the microscope. Only the intact cells were considered to count the chromosome number, while modal number was considered to ascertain the somatic chromosome number.

### 4.5. Total RNA Isolation and Library Preparation

Total RNA from the apical leaves of mature plants (six months old) of *O. basilicum* (OBP1) and *O. kilimandscharicum* (OKP2)*,* interspecific hybrid F1 and amphidiploids were isolated using a Spectrum Plant Total RNA Kit (Sigma Aldrich, St. Louis, MO, USA) according to the manufacturer’s protocol. The quantitative and qualitative analysis of the isolated RNA was performed using Nanodrop (Colibri, Baden Württemberg, Germany) and 1% agarose gel electrophoresis and an Agilent 2100 Bioanalyzer (Agilent Technologies, Santa Clara, CA, USA). An equal amount of RNA from for OBP1, OKP2, F1 and Amphid2 plants were used to construct and sequence RNA-seq libraries. The Illumina HiSeq 2000 platform was used for sequencing RNA-seq library, generating 144, 127, 139 and 135 Mbp of 101 bp paired-end reads for OBP1, OKP2, F1 and Amphid2 plants, respectively. All the contaminants and low-quality reads were removed for obtaining processed data. For the evaluation of the quality of raw reads and filtering high quality reads, a NGSQC Toolkit was used.

### 4.6. De Novo Assembly and Functional Annotation

The *de novo* transcriptome assembly of high-quality reads was carried out by the Trinity Assembler (Trinityrnaseq-2.0.6) using a 200 bp minimum contig length and 2 bp minimum count for K-mers as per the Inchworm algorithm for the assembly of primary transcriptomes. CD-HIT-EST (v4.6.1) having 90% coverage and identity 80 and the TransImprove-2.0.1 (Bionivid) tool were used for the final transcriptome assembly. Validated and ameliorated reads with an average depth ≥ 5 and coverage ≥ 70% were used as an input for CD-Hit-EST. The basic local alignment search tool program (Blast X; E value ≤ 0.001) was used against the NCBI non-redundant (NR) protein database for the annotation of final transcriptome assembly.

### 4.7. Differential Gene Expression (DGE) Analysis

Determination of differentially expressed genes (DEGs) between progenies and their progenitors was performed using the DESeq R Package [58]. The DESeq package provides methods to test for differential expression using the negative binomial distribution. Read count of the transcripts was used as an input for the analysis of differentially expressed genes. For the estimation of genotype-specific transcriptional differences, the following pair-wise contrasts were carried out: OBP1 vs. OKP2, OBP1 vs. F1, OBP1 vs. Amphid2, OKP2 vs. F1, OKP2 vs. Amphid2 and F1 vs. Amphid2 (Tables S8–S13; Table S15). Genes with a fold change value greater than zero were up-regulated and less than zero were down-regulated. For the analysis of differentially expressed genes in OBP1, OKP2, F1 and Amphid2, transcripts having a log_2_ fold change −1≥ and ≤1 were considered. However, up- and down-regulated genes with an FDR adjusted *p*-value < 0.05 were considered statistically significant.

### 4.8. Quantitative RT-PCR for Gene Expression Analysis

To validate the results of RNA-seq data, qRT-PCR was conducted as per the protocol described by Rastogi et al., 2016 [35]. Total RNA (2 μg) isolated (Spectrum Platnt Total RNA Kit, Sigma Aldrich, St. Louis, MO, USA) from all genotypes was used for the synthesis of cDNA (cDNA synthesis Kit, Thermo Scientific, Waltham, MA, USA). This cDNA was taken as the template for the quantification of relative mRNA levels following SYBR green chemistry (Maxima SYBR Green 2 x PCR Master Mix, Thermo Scientific, Waltham MA, USA), performed on a Fast real-time PCR system (7900HT applied Biosystems, Waltham, MA, USA). The gene sequences were selected from the RNA-seq data of all four *Ocimum* genotypes used in this study. The PCR reactions for each genotype of *Ocimum* were run in triplicate and the mean value of each sample was statistically analyzed. Actin was used as an endogenous control. Primers for qRT-PCR were designed through Primer express software (Agilent technologies, Santa Clara, CA, USA; Table S14).

### 4.9. Gene Ontology Enrichment Analysis

Differentially expressed genes were analyzed using KEGG Orthology-Based Annotation System KOBAS for the determination of significantly enriched pathways. The statistically enriched DEGs were considered significant with a false discovery rate (FDR) < 0.05. Further, AgriGO (http://bioinfo.cau.edu.cn/ (accessed on 19 May 2021)) was used to summarize and visualize Gene Ontology terms using *Arabidopsis thaliana* as a background species. The color of circles indicates the enrichment in the form of log10 *p*−value from the AgriGO results (blue lower, red higher).

### 4.10. Oil Analysis

For relative oil analysis, 100 g of fresh leaves of interspecific hybrid F1 and amphidiploids were hydro-distilled in the Clevenger’s apparatus. The oil samples of both plants were collected into the micro-centrifuge tubes (MCTs) separately. Then, 1 µL of dehydrated (using anhydrous sodium sulphate) oil diluted with hexane in the ration of 1:10 was injected into the GC-MS system (MSD 7890A, Agilent Technologies) equipped with an autosampler, HP-5MS column and 7977A mass detector. The oil samples were run in split-less mode as described by Akhtar et al., 2017 [59]. All the samples were run in three biological replicates and analyzed statistically (±standard deviation). Mass spectra acquisition was carried out in scan mode and analyzed with the help of Mass hunter workstation software (Agilent technologies) by comparing with the NIST11 library.

### 4.11. Biochemical Assays for Estimation of Total Chlorophyll, Total Lignin and Total Flavonoid Contents

Total chlorophyll content of both interspecific hybrid F1 and amphidiploids was calculated as described by Arnon, 1949 [60]. The absorbance of the pigment was estimated at 645 and 663 nm wavelength against 80% acetone. Three biological replicates were taken for the analysis, and absorbance of each replicate was repeated thrice.

Total lignin content from the mature leaves of three biological replicate samples of interspecific hybrid F1 and amphidiploids were estimated using the method as described by Kumar et al., 2016 [61].

Total flavonoid content was quantified from the dried samples of mature leaves of interspecific hybrid F1 and amphidiploids using the Dowd method as illustrated by Sankhalkar and Vernekar, 2016 [62].

### 4.12. Statistical Measurement

Microsoft Office Excel 2007 was used for the calculation of mean values and standard error (SE). GraphPad prism software was used for the calculation of significant differences between the samples. *** *p* < 0.0001, ** *p* < 0.001 and * *p* < 0.05.

## 5. Conclusions

In this investigation, phenotypic and anatomical comparisons showed the existence of a developmental alteration between interspecific hybrid F1 and amphidiploids. The detection of gene expression alteration in interspecific hybrid F1 and amphidiploids clearly depicted the loss of expression, suppression and activation in interspecific hybrid F1 and amphidiploids. A lower amount of total lignin and flavonoid contents but higher amount of eugenol and methyl eugenol in interspecific hybrid F1 was due to the diversion of some flux towards the production of higher phenylpropenes in interspecific hybrid F1. Moreover, change in total chlorophyll content of interspecific hybrid F1 and amphidiploids was correlated with the up-regulation of the chlorophyll biosynthesis pathway in interspecific hybrid F1 and up-regulation of chlorophyll degradation pathway in amphidiploids. Furthermore, the decreased amount of total lignin also signifies its role in the developmental changes between interspecific hybrid F1 and amphidiploids. In addition, several differentially expressed genes related to stomatal patterning and development could be responsible for the higher density of stomata in interspecific hybrid F1, and a larger size of stomata in amphidiploids was identified. Further, expression analysis of many TFs putatively involved in the growth and development processes, such as *MYB*-like transcription factor *ETC3*, PREDICTED: trihelix transcription factor *GTL1* isoform X2, zinc finger protein *CONSTANS-LIKE 5* -like, etc., in interspecific hybrid F1 integrates the transcriptome data correlating the phenotypic differences in interspecific hybrid F1 and amphidiploids.

## Figures and Tables

**Table 1:** Phenotypic comparison of parents (OBP1 and OKP2), interspecific hybrid F1 and amphidiploids.

**Trait**	OBP1	OKP2	F1	Amphid2
**Plant height (cm)**	87.40 ± 7.80	110.00 ± 8.9 ***	112.70 ± 6.24 ***	101.50 ± 9.30 **
**Leaf area (cm^2^)**	7.61 ± 0.61	6.85 ± 0.45 *	3.50 ± 0.18 ***	9.63 ± 0.75 ***
**Stem diameter (cm)**	3.40 ± 0.60	3.47 ± 0.50	2.92 ± 0.40	6.02 ± 0.70 ***
**Length of inflorescence (cm)**	12.06 ± 1.70	13.30 ± 1.20	24.30 ± 1.90 ***	17.60 ± 1.90 ***
**Trichome density**	42.20 ± 2.87	29.80 ± 1.18 ***	62.30 ± 1.87 ***	26.20 ± 0.54 ***
**Trichome length (µm)**	69.85 ± 2.75	72.54 ± 1.87 *	70.92 ± 1.32	88.30 ± 2.71 ***
**Stomata length (µm)**	12.44 ± 0.65	13.00 ± 1.08	7.90 ± 0.61 ***	21.20 ± 1.08 ***
**Oil yield (mg/100 g leaves)**	434.67 ± 25.98	307.33 ± 31.22 **	411.33 ± 43.57	488.67 ± 22.10

*** *p* < 0.0001, ** *p* < 0.001 and * *p* < 0.05 for one-way ANOVA (Bonferroni’s Multiple Comparison Test).

## Data Availability

The NGS data of the present investigation were submitted to the NCBI SRA database under the bioproject ID PRJNA520976.

## Supplementary Materials

The following supporting information can be downloaded at: https://www.mdpi.com/article/10.3390/plants11141873/s1, Table S1: Assembly Report; Table S2: Annotation Summary; Table S3: DEGs co-regulated; Table S4: Transcripts related to general phenylpropanoid biosynthetic pathway; Table S5: Transcripts related to chlorophyll metabolism; Table S6: Transcripts related to stomatal patterning and development genes; Table S7: Transcription factor No.; Table S8: DGE OBP1 vs. OKP2.XLSX; Table S9: DGE OBP1 vs. F1. XLSX; Table S10: DGE OBP1 vs. Amphid2.XLSX; Table S11: DGE OKP2 vs. F1.XLSX; Table S12: DGE OKP2 vs. AMPHID2.XLSX; Table S13: DGE F1 vs. AMPHID2.XLSX; Table S14: List of primers used in Real-Time PCR; Table S15: FPKM; Figure S1A–D: Parent 1 (*Ocimum basilicum* OBP1), Parent 2 (*Ocimum kilimandscharicum* OKP2), interspecific hybrid F1 and Amphidiploid. (E) Inflorescence of Parent 1 (OBP1), Parent 2 (OKP2), interspecific hybrid F1 and Amphidiploid. (F–I) Representation of stem diameter of Parent 1 (OBP1), Parent 2 (OKP2), interspecific hybrid F1 and Amphidiploid; Figure S2A–D: Microscopic view showing trichome density of Parent 1 (OBP1), Parent 2 (OKP2), interspecific hybrid F1 and Amphidiploid at 40X; Figure S3A: Venn diagram representing number of differentially expressed genes in F1 vs. Amphid2. Figure S3B KEGG Orthology (KO) classification: Bar graph shows the number of transcripts among different pathway categories; Figure S4A: Interactive graph showing Gene Ontology term in biological process category of differentially expressed genes in F1 vs. Amphid2. Figure S4B: Interactive graph showing Gene Ontology term in molecular process category of differentially expressed genes in F1 vs. Amphid2. Color of bubble shows *p*-value and size of bubble shows frequency of the GO terms. Highly similar GO terms are linked by the edges in the graph; Figure S5: GC-MS profiling of oils (extracted from the 100 g leaves of OBP1, OKP2, F1 and Amphid2 plants) showing peaks of eugenol and methyl eugenol in OBP1, F1 and Amphid2 but not in OKP2. Dotted square box in red color is showing the peaks of eugenol and dotted square box in black color is showing the peaks of methyl eugenol.
