# Supplementary material for: Altered Developmental and Metabolic Gene Expression in Basil Interspecific Hybrids
Source: Plants (Basel). 2022 Jul 18;11(14):1873. doi: 10.3390/plants11141873 (PMC9321874; doi:10.3390/plants11141873)
Supplement: Supplementary file 1 [file plants-11-01873-s001.zip › Supplementary Plants.pdf]

Altered developmental and metabolic gene expression in Basil interspecific hybrids

Saumya Shah[1], Shubhra Rastogi[1], Divya Vashisth[1], Prashant Kumar Rout[2], Raj Kishori Lal[3], Umesh Chandra Lavania[4], Ajit Kumar Shasany[1,5]*

[1]Biotechnology Division, CSIR-Central Institute of Medicinal and Aromatic Plants, Lucknow 226015, India

[2]Department of Phytochemistry, CSIR-Central Institute of Medicinal and Aromatic Plants, Lucknow 226015, India

[3]Department of Genetics and Plant breeding, CSIR-Central Institute of Medicinal and Aromatic Plants, Lucknow 226015, India

[4]Department of Botany, University of Lucknow, Lucknow 226007, India

[5]ICAR-National Institute for Plant Biotechnology (NIPB), Pusa Campus, New Delhi 110012, India

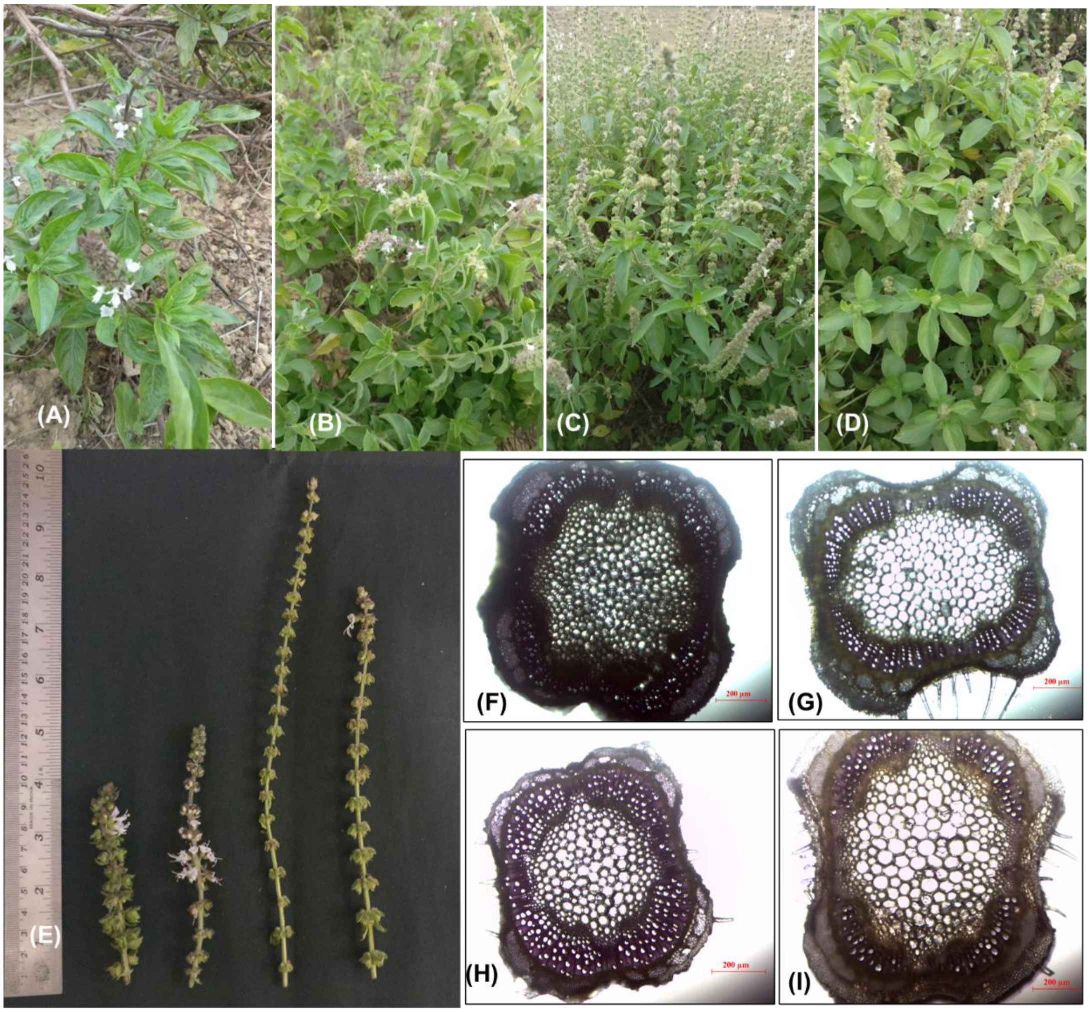

Figure S1 A-D Parent 1 (*Ocimum basilicum* OBP1), Parent 2 (*Ocimum kilimandscharicum* OKP2), interspecific hybrid F1 and amphidiploid. **(E)** Inflorescence of Parent 1 (OBP1), Parent 2 (OKP2), interspecific hybrid F1 and amphidiploid. **(F-I)** Representation of stem diameter of Parent 1 (OBP1), Parent 2 (OKP2), interspecific hybrid F1 and amphidiploid.

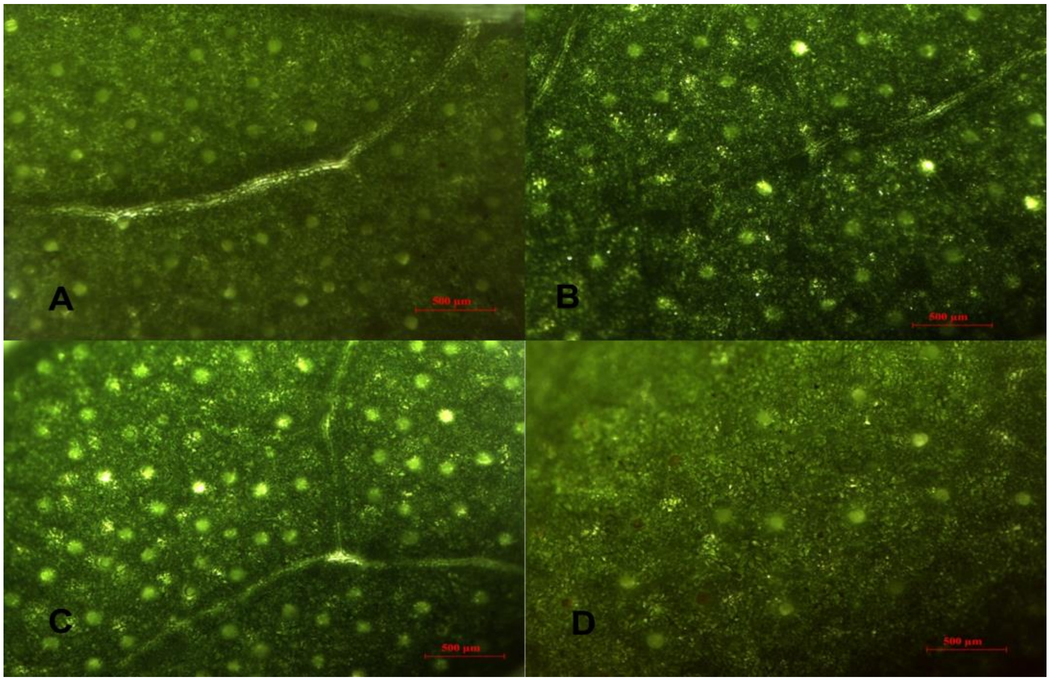

Figure S2 A-D Microscopic view showing trichome density of Parent 1 (OBP1), Parent 2 (OKP2), interspecific hybrid F1 and amphidiploid at 40X.

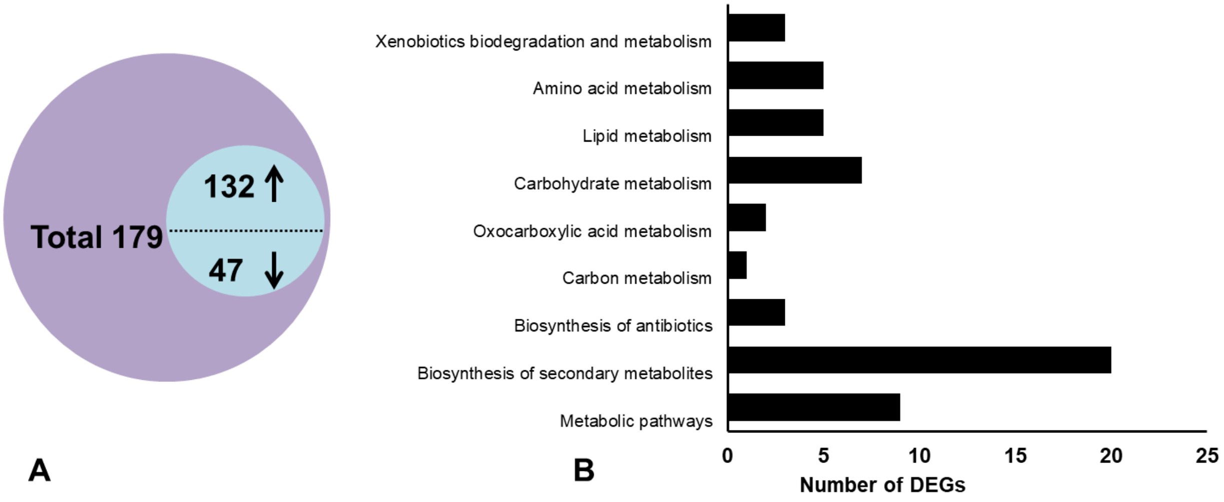

Figure S3A Venn diagram representing number of differentially expressed genes in F1*vs*. Amphid2. **Figure S3B** KEGG Orthology (KO) classification: Bar graph shows the number of transcripts among different pathway categories.

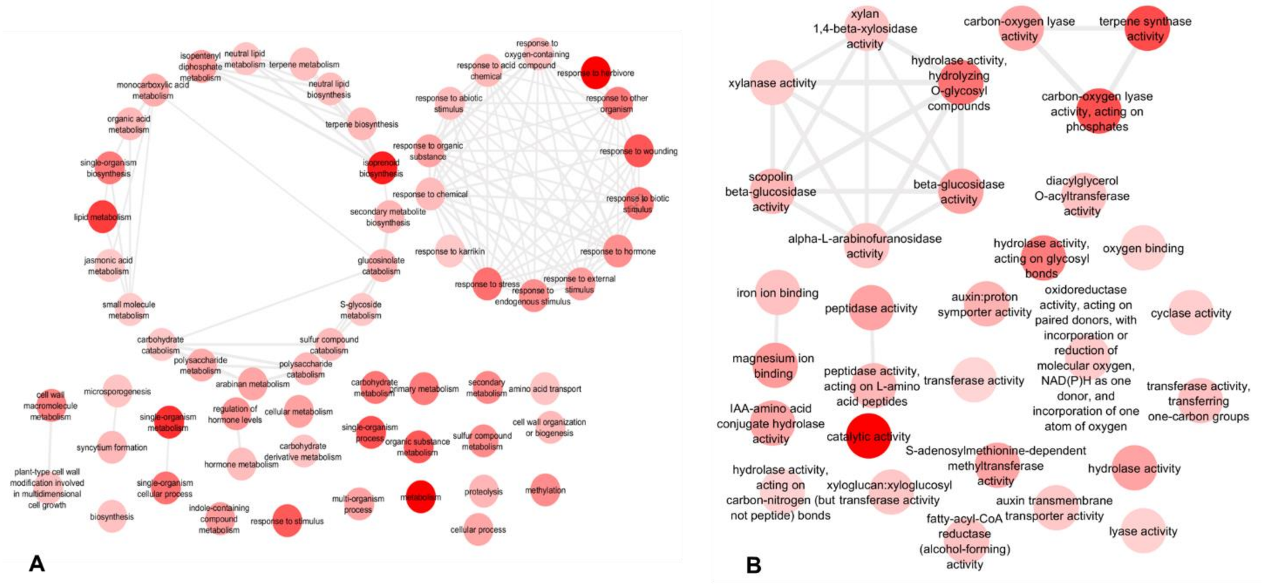

Figure S4A Interactive graph showing Gene Ontology term in biological process category of differentially expressed genes in F1 vs Amphid2 **S4B** Interactive graph showing Gene Ontology term in molecular process category of differentially expressed genes in F1 vs. Amphid2. Color of bubble shows P-value and size of bubble shows frequency of the GO terms. Highly similar GO terms are linked by the edges in the graph.

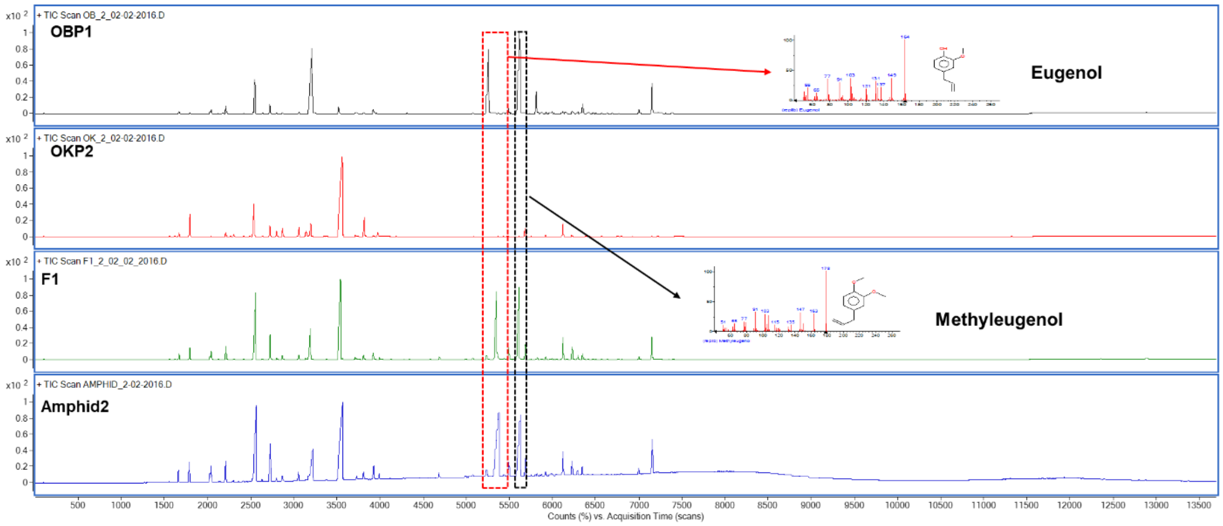

Figure S5 GC-MS profiling of oils (extracted from the 100g leaves of OBP1, OKP2, F1 and Amphid2 plants) showing peaks of eugenol and methyl eugenol in OBP1, F1 and Amphid2 but not in OKP2. Dotted square box in red color is showing the peaks of eugenol and dotted square box in black color is showing the peaks of methyl eugenol.
